# Supplementary material for: Exploring Rural Adolescents’ Dietary Diversity and Its Socioeconomic Correlates: A Cross-Sectional Study from Matlab, Bangladesh
Source: Nutrients. 2020 Jul 26;12(8):2230. doi: 10.3390/nu12082230 (PMC7468778; doi:10.3390/nu12082230)
Supplement: Supplementary file 1 [file nutrients-12-02230-s001.zip › nutrients-853089-s/Figure S1.pdf]

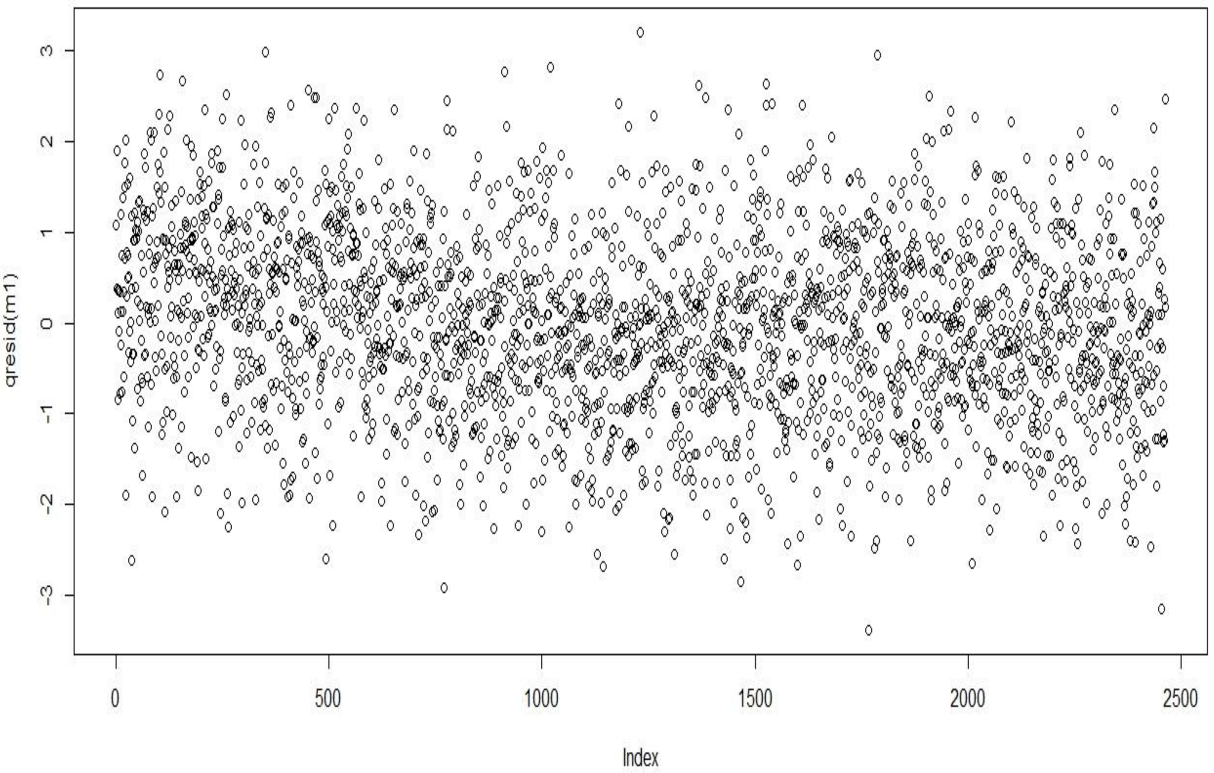

Figure S1. Plot of quantile residuals for the fitted binary logistic regression (m1 indicates the model and qresid stands for quantile residulas).
